# Disruption of the Golgi protein *Otg1* gene causes defective hormone secretion and aberrant glucose homeostasis in mice

**DOI:** 10.1186/s13578-016-0108-4

**Published:** 2016-06-10

**Authors:** Guangxue Wang, Rongbo Li, Ying Yang, Liang Cai, Sheng Ding, Tian Xu, Min Han, Xiaohui Wu

**Affiliations:** State Key Laboratory of Genetic Engineering and National Center for International Research of Development and Disease, Institute of Developmental Biology and Molecular Medicine, Collaborative Innovation Center for Genetics and Development, School of Life Sciences, Fudan University, Shanghai, 200433 China; Howard Hughes Medical Institute, Department of Genetics, Yale University School of Medicine, New Haven, CT 06536 USA; Howard Hughes Medical Institute, Department of Molecular, Cellular and Developmental Biology, University of Colorado, Boulder, CO 80309 USA

**Keywords:** *Otg1*, Hypoglycemia, Hypoinsulinemia, Vesicle trafficking

## Abstract

**Background:**

Concerted hormone secretion is essential for glucose homeostasis and growth. The oocyte testis gene 1 (*Otg1*) has limited information in mammals before. Human OTG1 has been identified as an antigen associated with cutaneous T cell lymphoma, while worm *Otg1* is recently reported to be a vesicle trafficking regulator in neurons. To understand the physiological role of *Otg1* and its potential relation to hormone secretion, we characterized a mutation caused by the *piggyBac* transposon (*PB*) insertion in mice.

**Results:**

Oocyte testis gene 1 encodes a Golgi localized protein that is expressed with a broad tissue distribution in mice. The *PB* insertion effectively blocks *Otg1* expression, which results in postnatal lethality, growth retardation, hypoglycemia and improved insulin sensitivity in mice. *Otg1* mutants exhibit decreased levels of insulin, leptin and growth hormone in the circulation and reduced hepatic IGF-1 expression. Decreased expression of *Otg1* in pituitary GH3 cells causes reduced grow hormone expression and secretion, as well as the traffic of the VSVG protein marker.

**Conclusions:**

Our data support the hypothesis that *Otg1* impacts hormone secretion by regulating vesicle trafficking. These results revealed a previously unknown and important role of *Otg1* in hormone secretion and glucose homeostasis in mammals.

**Electronic supplementary material:**

The online version of this article (doi:10.1186/s13578-016-0108-4) contains supplementary material, which is available to authorized users.

## Background

Glucose is the key source for energy production in mammals. Under normal physiological conditions, the blood glucose level is well regulated by concerted actions of the pancreas, liver, adipose tissue, muscle and brain [1, 2]. Abnormal glucose homeostasis would result in hyperglycemia or hypoglycemia. Hyperglycemia is the characteristic condition of diabetes, which has becoming a rapidly growing health threat in modern society [3]. Chronic hyperglycemia causes glycation of proteins or lipids, which causes many of the long-term complications in diabetic patients [4]. In contrast, since glucose supplies almost all the energy for the brain, hypoglycemia may quickly cause loss of consciousness or even death.

Peptide hormones such as insulin, glucagon, growth hormone and IGF-1 play critical roles in regulating glucose homeostasis [5–9]. Being expressed, peptide hormones are packaged in vesicles at the trans-Golgi network (TGN), transported on microtubules toward the plasma membrane and loaded onto an actin/myosin system for distal transport through the actin cortex to just below the plasma membrane. After tethered there, a subpopulation of vesicles are docked and primed to become the readily-releasable pools [10, 11]. Upon stimulation, these vesicles in the readily-releasable pool would immediately fuse to the plasma membrane to release the contents. This process is essential for activity-dependent hormone secretion to mediate various endocrinological functions. Despite of many identified proteins involved in vesicle budding, trafficking, tethering/docking and cargo secretion, the molecular mechanisms and molecules participating peptide hormone secretion remain to be explored.

The oocyte-testis gene 1 (*Otg1*) was originally identified in the RIKEN Mouse Gene Encyclopedia Project [12]. The full-length transcript has 16 exons that encode a 917-amino acid peptide. The OTG1 protein has several coiled-coil domains that occupy almost half of the peptide. Other than these, no functional motifs have been predicted in OTG1 [13]. The human homologue of *Otg1* encodes the protein that has been recognized as a cutaneous T-cell lymphoma (CTCL) associated antigen [14]. Recently, the *C. elegans* homologue of *Otg1* has been reported as a vesicle trafficking regulator in neurons [15]. Here we report that mouse *Otg1* encodes a protein with prominent Golgi localization. Loss of *Otg1* results in postnatal lethality, aberrant glucose homeostasis and defective hormone secretion in mice. These results revealed an unknown role of *Otg1* in participating hormone secretion and metabolic regulation in mammals.

## Results

### Disruption of *Otg1* results in postnatal lethality and growth retardation in mice

**Fig. 1:**
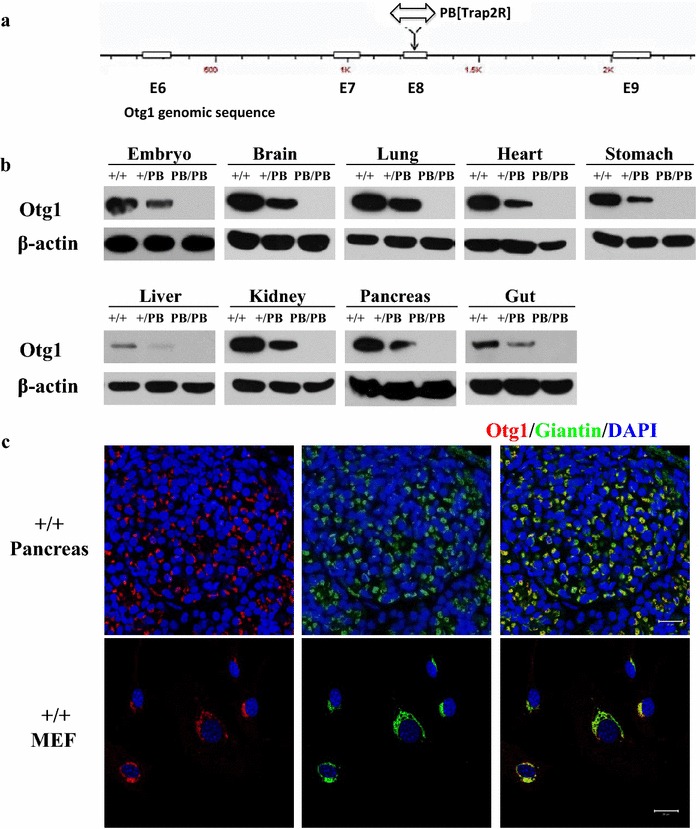
A PB insertion effectively abolished *Otg1* expression. **a** Schematic representation of the genomic sequence flanking *PB* insertion site in *Otg1*. *White box*: exon. **b** Western blot did not detect OTG1 expression in either E13.5 *Otg1* ^*PB/PB*^ embryos or tissues of P1 *Otg1* ^*PB/PB*^ mice. **c** Immunofluorescence staining showed co-localization of OTG1 protein and Giantin in both pancreatic tissues of MEFs from wild-type mice

We identified an *Otg1* mutant in a screen for mice bearing metabolic defects [16]. The mutant carries a *piggyBac* transposon (*PB*) insertion in the eighth exon of *Otg1* (*Otg1*^*PB*^) that effectively disrupts gene expression (Fig. 1a). In wild-type animals, *Otg1* proteins can be readily detected in various organs such as the brain, heart, lung, stomach, liver, kidney, pancreas and gut. In tissues from homozygous mutants, *Otg1* expression is no longer detectable (Fig. 1b). Similar changes were also observed by immunofluorescence staining in pancreatic cells and embryonic fibroblasts (MEFs) with different genotypes (Fig. 1c and Additional file 1: Figure S1). Similar as that of the *C. elegans* homologue, immunofluorescence staining in wild-type pancreatic cells and MEFs revealed co-localization of OTG1 proteins with the Golgi compartment marker Giantin, confirming a Golgi localization of OTG1 in mice (Fig. 1c) [15, 17].

**Fig. 2:**
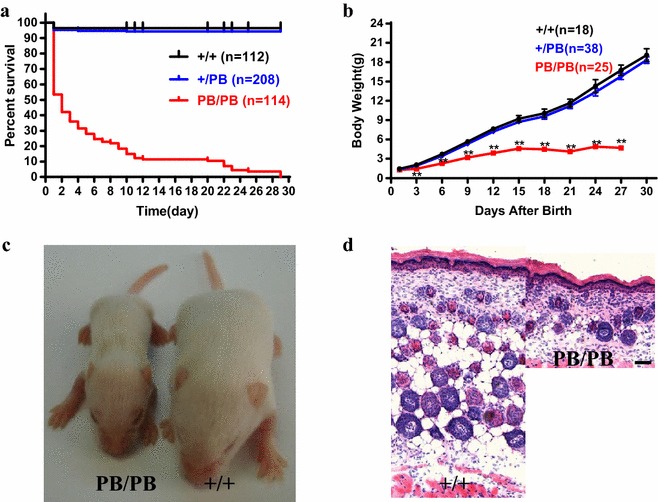
Postnatal lethality and growth retardation in *Otg1* mutant mice. **a** Survival curves of *Otg1* ^*PB/PB*^ (n = 114), *Otg1* ^*PB/*+^ (n = 208) and wild-type (n = 114) littermates. **b** Body weight curves of *Otg1* ^*PB/PB*^ (n = 25), *Otg1* ^*PB/*+^ (n = 38) and wild-type (n = 18) littermates. **c** Representative image showing the body size difference between an *Otg1* ^*PB/PB*^ and a wild-type mouse at P11. **d** Representative image of the hypodermis histological section of P4 *Otg1* ^*PB/PB*^ and *Otg1* ^+*/*+^ mice, showing severe lipodystrophy of the mutant

Oocyte testis gene 1^*PB/PB*^ animals were born in consistent with a Mendelian pattern of inheritance (Additional file 2: Table S1). However, 46.5 % (53/114) homozygous *Otg1* mutants could not survive throughout the first day after birth (P1), while others gradually died within the next 30 days. In contrast, 96.4 % (108/112) wild-type and 94.2 % (196/208) heterozygous littermates kept alive at the age of one month (Fig. 2a). The external morphology of mutants died at P1 was apparently normal. In contrast, the most obvious morphological changes of other dead mutants were their small sizes (Fig. 2c). Further analysis showed that *Otg1*^*PB/PB*^ individuals had comparable body weight with their littermates both at the end of fetal development (embryonic day 18.5, Additional file 3: Figure S2C) and at birth (Fig. 2b). Soon after that, the survivors suffered from severe growth retardation. The body weight of *Otg1*^*PB/PB*^ mice increased much slower than that of the wild-type and heterozygous littermates. In fact, homozygous *Otg1* mutants always exhibit lipohypotrophy and usually died before the body weight reaches 5 grams (Fig. 2b, d).

### *Otg1* mutation leads to impaired glucose homeostasis

We then explored pathophysiological alterations that may lead to postnatal lethality and growth retardation in *Otg1*^*PB/PB*^ mice. Alcian blue-alizarin red staining revealed normal skeleton structures in *Otg1*^*PB/PB*^ mutants (Additional file 3: Figure S2A). This result, along with the normal body weight of newborn animals, suggests that severe embryonic developmental defects, such as abnormal pattern formation, shall not be accounted for postnatal lethality and growth retardation. We often observed milk in the stomach of *Otg1*^*PB/PB*^ pups, suggesting that feeding failure is unlikely the reason for growth retardation and lethality (Additional file 3: Figure S2B).

**Fig. 3:**
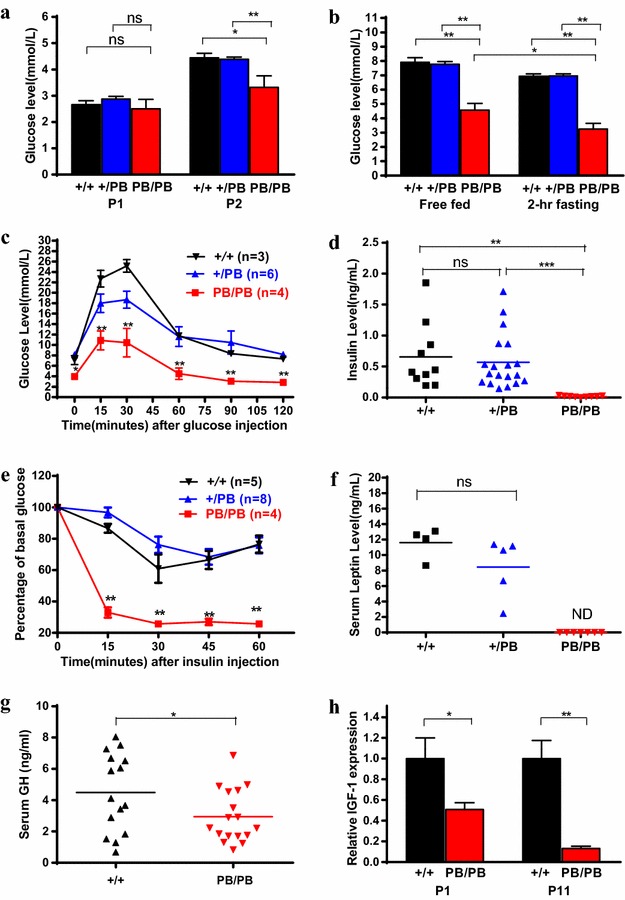
Impaired glucose homeostasis in *Otg1* mutant mice. **a** Blood glucose levels of newborn *Otg1* ^*PB/PB*^ (P1: n = 13; P2: n = 10), *Otg1* ^*PB/*+^ (P1: n = 30; P2: n = 30) and wild-type (P1: n = 16; P2: n = 11) littermates. **b** Blood glucose levels of free fed or 2-hour starved *Otg1* ^*PB/PB*^ (n = 10), *Otg1* ^*PB/*+^ (n = 19) and wild-type (n = 10) littermates at P11. **c** Intraperitoneal glucose tolerance test (IPGTT) performance of *Otg1* ^*PB/PB*^ (n = 4), *Otg1* ^*PB/*+^ (n = 6) and wild-type (n = 3) littermates at P11. **d** Serum insulin levels of *Otg1* ^*PB/PB*^ (n = 10), *Otg1* ^*PB/*+^ (n = 19) and wild-type (n = 10) littermates at P11. **e** Insulin tolerance test (ITT) results of *Otg1* ^*PB/PB*^ (n = 4), *Otg1* ^*PB/*+^ (n = 8) and wild-type (n = 5) littermates at P11. **f** Serum leptin levels of *Otg1* ^*PB/PB*^ (n = 7), *Otg1* ^*PB/*+^ (n = 5) and wild-type (n = 4) littermates. **g** Serum growth hormone levels of *Otg1* ^*PB/PB*^ (n = 17) and wild-type (n = 15) littermates. **h** Relative IGF-1 expression in *Otg1* ^*PB/PB*^ (P1: n = 5; P2: n = 4) and wild-type (P1: n = 3; P11: n = 5) littermates. *p < 0.05, **p < 0.01, ***p < 0.005

Given the critical role of glucose in supporting growth, we examined glucose homeostasis in mutant mice. We observed progressively developed hypoglycemia in *Otg1* mutants. The blood glucose level in free–fed *Otg1*^*PB/PB*^ mice was similar as that in the wild-type and heterozygous littermates at birth, then decreased to approximately 25 % blow normal within two days and further dropped to 58 % of that in the wild-type at the age of 11 days (P11) (Fig. 3a, b). Fasted blood glucose levels of P11 *Otg1*^*PB/PB*^ mice were only 47 % of that in the wild-type mice. In intraperitoneal glucose tolerance test (IPGTT), the blood glucose level of P11 *Otg1*^*PB/PB*^ mice changed with the same tendency as that of the wild-type or heterozygous mice, but kept to be approximately 60 % lower at each time point (Fig. 3c). In addition to hypoglycemia, we recorded extremely low level of serum insulin and elevated insulin sensitivity in the mutants. Compared with 0.66 and 0.57 ng/ml of serum insulin detected in wild-type and heterozygous littermates, respectively, ELISA analysis revealed an average insulin concentration of 0.02 ng/ml in P11 homozygotes (Fig. 3d). In the insulin tolerance test (ITT), the blood glucose level of P11 *Otg1*^*PB/PB*^ mice dropped more rapidly than that of the wild-type and heterozygous littermates. In fact, it decreased to a level too low to be detected within 30 min (Fig. 3e). Finally, we observed hypoleptinemia in *Otg1* mutants. Consistent with lipodystrophy, *Otg1*^*PB/PB*^ mice had circulating leptin below detectable level (<0.2 ng/ml) at P11 (Fig. 3f).

Growth retardation, hypoinslulinemia, hypoglycemia and increased insulin sensitivity have been reported in mice with defective growth hormone receptors (GHRs) [7, 9]. This raises the possibility that growth hormone (GH) signaling is aberrant in *Otg1*^*PB/PB*^ mice. We measured the expressions of GH and its downstream mediator IGF-1. Compared with those of the wild-type littermates, ELISA revealed approximately 35 % reduction of serum GH levels in P11 *Otg1*^*PB/PB*^ mice, while real-time RT-PCR detected 50 and 87 % decrease of hepatic *IGF*-*1* expression in P1 and P11 mutants, respectively (Fig. 3g, h). Taken together, the results above suggest that disruption of *Otg1* leads to impaired glucose homeostasis in mice.

### *Otg1* mutation leads to aberrant vesicle trafficking

**Fig. 4:**
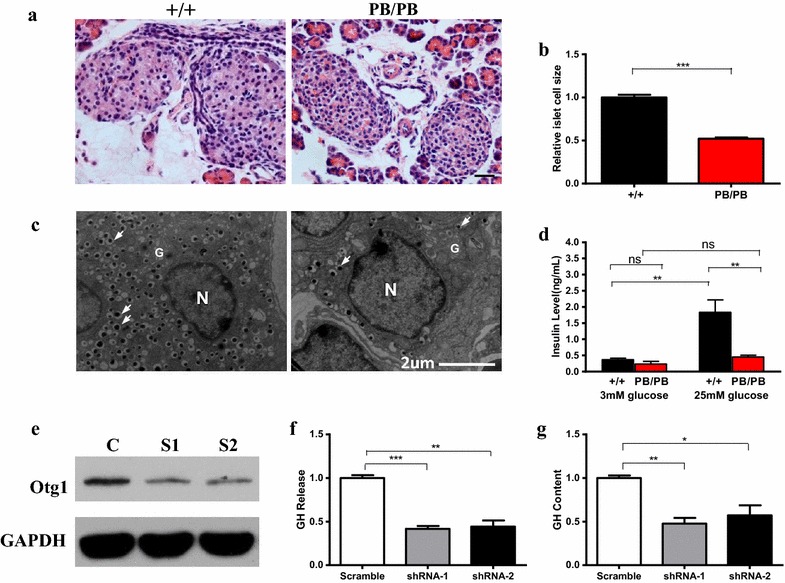
Decreased *Otg1* expression leads to impaired hormone secretion. **a** H&E staining of pancreas from P11 *Otg1* ^*PB/PB*^ and wild-type littermates. **b** Relative pancreas islet cell sizes of P11 *Otg1* ^*PB/PB*^ (n = 31) and wild-type (n = 20) littermates. **c** Scanning EM of islet cells of P6 *Otg1* ^*PB/PB*^ and wild-type littermates. *White arrows* indicate insulin granules. N: nucleus. G: Golgi. **d** Insulin levels of glucose stimulated insulin secretion (GSIS) after isolation of islets isolated from *Otg1* ^*PB/PB*^ (n = 7) and wild-type (n = 8) littermates at P1. **e** Western blot showed the decreased *Otg1* expression in GH3 cells transfected with small hairpin RNAs, but not scramble shRNAs. GAPHD serves as the loading control. **f** Released growth hormone levels in *Otg1* knockdown cells compared with cells transfected with scramble shRNAs, which is arbitrary set as 1. **g** Growth hormone levels in *Otg1* knockdown cells compared with cells transfected with scramble shRNAs, which is arbitrary set as 1. *p < 0.05, **p < 0.01, ***p < 0.005

Given its Golgi localization and the reported role of the *C. elegans* homologue in neurons [15], *Otg1* is likely to be involved in vesicle trafficking, a process that is critical for protein hormone secretion in mammals. Consistent with this predicted role, we found *Otg1*^*PB/PB*^ mice had islet cells 48 % of the sizes of their wild-type littermates (Fig. 4a, b). This is likely the consequence of defective vesicle trafficking rather than the result of smaller body size, since *Otg1*^*PB/PB*^ hepatocytes are of similar sizes as those of the *Otg1*^+*/PB*^ animals (Additional file 4: Figure S3). Electron microscopy also revealed a greatly reduced number of insulin granules in the cytoplasm of mutant β cells (Fig. 4c). To mask the possible effect on insulin secretion from reduced GH signaling in vivo, we isolated islets from newborn mutants and examined their response to glucose challenge by measuring secreted insulin in the culture medium. There was no significant difference between *Otg1*^*PB/PB*^ and the wild-type islets when they were provided with basal level (3 mM) of glucose. However, when challenged by 25 mM of glucose for 1 h, the insulin concentration in the culture medium of *Otg1*^*PB/PB*^ islets was only 25 % of that of the wild-type islets (Fig. 4d). Altogether, these results suggest that the smaller islet cells are likely the result of defective vesicle trafficking caused by the *Otg1* mutation.

The role of *Otg1* in hormone secretion was further confirmed in rat pituitary GH3 somatolactotropes, a popular model to study GH secretion [18]. We first knocked down *Otg1* expression with small hairpin RNAs (shRNAs), then measured GH released into the medium within a period of two hours. Compared with cells transfected with scramble shRNAs, shRNA-1 and shRNA-2 transfected cells produced 55 and 58 % less *Otg1* proteins, respectively (Fig. 4e). As expected, shRNA-1 transfection resulted in a reduction of GH content and secretion by 52 and 58 %, respectively, while shRNA-2 transfection resulted in a reduction of GH content and secretion by 43 and 56 %, respectively (Fig. 4f, g).

**Fig. 5:**
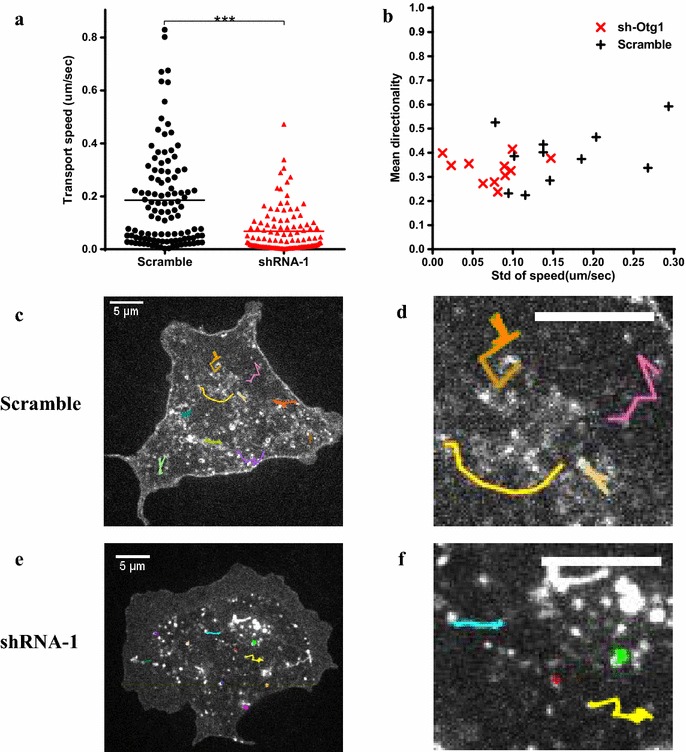
Blocked intracellular transport in *Otg1* knockdown cells. GH3 cells were co-transfected with VSVG-mEmerald and *Otg1* shRNA or scramble shRNA. **a** *Scatter plot* showing decreased transport speed of VSVG-mEmerald vesicles in *Otg1* knockdown (*red triangles*) and scramble shRNA treated (*black dots*) GH3 cells. Eleven cells were scored for each group. Ten vesicles were randomly picked from each cell. **b** Scatter plot showing *Otg1* knockdown resulted in decreased standard deviation of transport speed (std speed) and unaltered directionality of VSVG-mEmerald vesicles in each cell. Data of vesicles selected in (**a**) were used for the calculation. **c**, **d** A representative image (**c**) and its close-up (**d**) showing tracked movement of 10 VSVG-mEmerald vesicles (*lines in color*) in scramble shRNA treated GH3 cells. **(e**, **f)** A representative image (**e**) and its close-up (**f**) showing tracked movement of 10 VSVG-mEmerald vesicles (*lines in color*) in *Otg1* knock down GH3 cells. *Scale bars* 5 μm. *p < 0.05, **p < 0.01, ***p < 0.005

Decreased hormone secretion in both mouse islet and rat GH3 suggested a critical role of *Otg1* in hormone secretion in mammals. To monitor the effect of *Otg1* on intracellular transport, we used VSVG-mEmerald, a fluorescent reporter that translocates from endoplasmic reticulum to the plasma membrane via the Golgi apparatus at 37 °C [19]. Live-cell imaging showed that the transportation of VSVG-mEmerald was significantly blocked in *Otg1* knockdown GH3 cells (Additional file 5: Video S1 and Additional file 6: Video S2). The average transport speed of VSVG-mEmerald vesicles (n = 110) was 0.068 μm/sec in *shRNA*-*1* treated cells, but 0.186 μm/sec in scramble shRNA treated cells (Fig. 5a). Standard deviations of the transport speed in each *shRNA*-*1* treated cell (n = 11) were also decreased (Fig. 5b and Additional file 7: Figure S4). These results suggest that *Otg1* is required for vesicle trafficking in mammalian cells.

## Discussion

In the present study, we have shown that *Otg1* encodes a Golgi protein that is required for normal vesicle trafficking in mammalian cells. Disruption of *Otg1* results in growth retardation and postnatal lethality in mice. Various abnormalities related to glucose homeostasis, such as hypoinslulinemia, hypoglycemia, increased insulin sensitivity, decreased serum growth hormone level and reduced hepatic IGF-1 expression, could be observed in mutant animals before death. These results revealed an unknown function *Otg1* in metabolic regulation.

Oocyte testis gene 1 is ubiquitously expressed with a prominent Golgi localization. Almost half of the peptide sequence is occupied by coiled-coil regions with short interruptions. OTG1 is a conserved protein during evolution and the human homologue has been identified as a tumor antigen. These features are reminiscent of those of the golgin coiled-coil proteins, which are known as membrane and cytoskeleton tethers [20, 21]. Although the C-terminus of OTG1 lacks a transmembrane or a small GTPase interacting signal, which are usually presented in a typical goglin, OTG1 may still be involved in similar intracellular activities by serving as molecular partners of typical golgins. Under this scenario, OTG1 may be involved not only in capturing incoming vesicles, but also in providing specificity to the tethering step.

Hypoglycemia is normal during the first hours of mammalian life. However, prolonged neonatal hypoglycemia would cause long-term neuronal deficits [22]. In contrast to extensively recognized hyperinsulinemic hypoglycemia, hypoinsunlinemic hypoglycemia is an extrememly rare condition in human. Limited cases of hypoinsunlinemic hypoglycemia are usually related to impaired insulin signaling pathway. For example, a hyperactive mutation of *AKT*2, the gene required for insulin-induced translocation of GLUT4 to the plasma membrane, caused hypoinsunlinemic hypoglycemia in four patients [23, 24]. On the other hand, non-islet cell tumor-induced hypoglycemia (NICTH) is caused by the secretion of incompletely processed precursors of IGF-II, which has an insulin-like hypoglycaemic activity [25]. We have shown that disruption of *Otg1* caused hypoinsunlinemic hypoglycemia in mice, which implies a possible role of *Otg1* mutations in human patients. The fact that *Otg1* mutation blocked vesicle trafficking also suggests a new etiology of this rarely observed disease condition. Examine other regulators of vesicle trafficking in human patients may identify more causative mutations of hypoinsunlinemic hypoglycemia in the future.

The mechanism through which *Otg1* modulates vesicle trafficking remains to be investigated. The *C. elegans* homologue of *Otg1* works as a partner of Rab-2 and Rund-1 in regulating neuronal vesicle trafficking [15]. However, mutations of the homologues of *Rab*-*2* (*Rab*-*2a and Rab*-*2b*) or *Rund*-*1* (*Rundc*-*1*) showed different phenotypes from that of *Otg1* mutants in mice [26, 27]. Unlike the *C. elegans* mutant, *Otg1*^*PB/PB*^ mice did not show gross behavioral defects. The observation that *Otg1*^*PB/PB*^ pups are capable of sucking milk suggests they may have normal neuronal functions (Additional file 3: Figure S2B). In addition, the human orthologue of *Otg1* is a tumor antigen. Thus, further studies of *Otg1* may not only shed light on the mechanisms of vesicle trafficking in mammals, but also contribute to the study of related diseases such as metabolic abnormalities or cancer.

## Conclusions

Our results revealed an essential role of *Otg1* in vesicle trafficking, which is critical for peptide hormone secretion, metabolic regulation and postnatal survival in mice.

## Methods

### Mice

All animal experiments were performed in accordance with protocols approved by the Animal Care and Use Committee of the Institute of Developmental Biology and Molecular Medicine (IDM), Fudan University. The *Otg1* mutant strain (H66eR12) was generated on the FVB/NJ background and maintained on 12/12-hour light/dark cycles. The *Otg1* mutation carried by H66eR12 was induced with a *piggyBac* transposon (*PB*) insertion in the eighth exon. Mapping information of the PB insertion in *Otg1* and the mutant genotyping protocol could be found from the PBmice database [16]. All assays were performed in a mixed population of both males and females.

### Metabolic assays

Blood glucose levels were analyzed with Glucometer Elite (LifeScan). For glucose tolerant tests (GTTs), animals were fasted two hours before receiving intraperitoneal injection of 20 % glucose saline solutions (2 g glucose per kg body weight). Tail vein blood was then sampled at 0, 15, 30, 60, 90 and 120 min after injection for blood glucose tests. For insulin tolerance tests (ITTs), 2-hour fasted mice received an intraperitoneal injection of insulin (Humulin, Lilly) (0.75 U/kg body weight), then had tail vein blood glucose levels measured at 0, 15, 30, 45 and 60 min later. ELISA was performed following the manufacturer’s protocol to measure serum insulin (Crystal Chem Inc.), leptin (Crystal Chem Inc.) and growth hormone (Millipore) concentrations. All samples were collected from female mice at the age of P11.

### Islet culture and glucose stimulated insulin secretion (GSIS)

Pancreatic islets were isolated by collagenase perfusion in situ, digested for 28 min and then purified by single layer histopaque (Sigma). Isolated islets from different mice were mixed and cultured in RPMI 1640 medium supplemented with 11 mM glucose, 7.5 % FCS and 10 mM HEPES (Sigma). For GSIS assay, islets were washed in PBS and incubated in a 96-well plate with glucose-free Krebs–Ringer bicarbonate (KRB) medium (125 mM NaCl, 4.74 mM KCl, 1 mM CaCl_2_, 1.2 mM KH_2_PO_4_, 1.2 mM MgSO_4_, 5 mM NaHCO_3_, 25 mM HEPES, pH 7.4, with 0.1 % BSA) at 37 °C for 30 min, then incubated in KRB containing 3 mM or 25 mM glucose at 37 °C for 30 min with 5 islets per well. The amount of insulin released into the incubation medium in each well was assayed using ELISA (Crystal Chem Inc.). At least 5 wells were examined for each genotype with each glucose concentration.

### Western blot

Protein extraction was prepared with the RIPA lysis buffer and quantified with the BCA Protein Assay Kit (Pierce). Equal amounts of samples were separated by SDS/PAGE, transferred onto PVDF membranes (Millipore) and immunoblotted following standard protocols. The antibodies used were: rabbit anti-OTG1 (Sigma HPA018019, 1:1000), mouse anti-GAPDH (KangCheng Biotech KC-5G4, 1:10,000) rabbit anti-β-actin (Santa Cruz sc-1616-R, 1:2000), goat anti-mouse IgG-HRP (Santa Cruz sc-2005, 1:5000) and goat anti-rabbit IgG-HRP (Santa Cruz sc-2004, 1:5000).

### Histology and immunohistochemistry

Frozen sections were prepared by fixing tissues overnight in 4 % paraformaldehyde, followed by cryoprotection in 30 % sucrose at 4 °C for two days and sectioning with given thickness for histological and immunofluorescence analysis. For morphological analysis, Section (5 μm) were stained with hematoxylin and eosin to have images acquired with a Leica DMRXA2 microscope. For immunofluorescence analysis, Section (6–8 μm) were treated following the standard protocol with following antibodies: rabbit anti-OTG1 (Sigma HPA018019, 1:1000), Alexa 488 conjugated rabbit-anti-Giantin (Covance A488-114L, 1:1000), rabbit anti-insulin (Santa Cruz sc-9168, 1:1000), goat anti-glucagon (Santa Cruz sc-7780, 1:1000), donkey anti-goat IgG-FITC (Chemicon AP180F, 1:2000), donkey anti-rabbit IgG-Cy3 (Millipore AP182C, 1:2000).

### Electron microscopy

Pancreas tissues were fixed in a fresh fixative solution consisting of 2 % glutaraldehyde and 4 % paraformaldehyde, postfixed with 1 % osmium tetroxide in phosphate buffer at 4 °C and dehydrated in ascending concentrations of methanol and propylenoxide before being embedded in Epoxy resin. Ultra-thin sections were prepared using a Reichert ultramicrotome, contrasted with uranyl acetate and lead citrate and examined under a Philips CM120 electron microscope.

### Cell culture and live imaging

Rat pituitary GH3 cells were cultured at 37 °C in a humidified atmosphere containing 95 % air and 5 % CO_2_. The culture medium was DMEM supplemented with 10 % fetal bovine serum, 100 U/ml penicillin and 100 mg/ml streptomycin. GH3 cells were transfected with small hairpin RNA (shRNA) constructs (Sigma-Aldrich) or the VSVG-mEmerald plasmid (modified from Addgene plasmid #31947) with Lipofectamine 2000 (Invitrogen). For growth hormone secretion assay, cells were transferred to serum free medium 48 h after transfection and incubated for 2 h before ELISA. Live cell confocal images were acquired 48 h after transfection, using spinning disk confocal scan head (CSU-X/M2 N, Yokogawa) attached to an inverted microscope (IX-81, Olympus) and an EMCCD camera (DU897BV, Andor) controlled by Micro-Manager software. Images (512 × 512 pixels, voxel size 0.0946 μm/pixel) were taken every 0.5 s for 400 frames. Live images were analyzed in NIH ImageJ with the MTrackJ plugin. VSVG containing vesicles (10/cell) were randomly selected in 11 *Otg1* knockdown cells and 11 scramble shRNAs treated cells. Directionality of each vesicle was defined as its real transport distance divided by linear distance between the start and end positions.

### Statistics

GSIS data were compared by two-way ANOVA analysis. All other data were compared by unpaired two-tailed Student’s *t* test. Results were shown as mean ± SEM. *P* < 0.05 was considered statistically significant.

## Additional files

10.1186/s13578-016-0108-4 *Otg1* expression is disrupted by the PB insertion. In contrast to Fig. 1c, immunofluorescence staining showed Giantin (green) but not *Otg1* (red) signals in pancreatic tissues and MEFs from *Otg1* ^*PB/PB*^ mice.
10.1186/s13578-016-0108-4 Genotypes of new born animals.
10.1186/s13578-016-0108-4 *Otg1* mutant mice have no severe developmental defects. **(A)** Alcian blue-alizarin red staining of P6 *Otg1* ^+*/PB*^ and *Otg1* ^*PB/PB*^ mice. **(B)** Representative image showing P6 wild-type and *Otg1* ^*PB/PB*^ littermates, with sucked milk in the stomach. **(C)** Average body weight of *Otg1* ^*PB/PB*^ (n = 6), *Otg1* ^*PB/*+^ (n = 15) and wild-type (n = 15) littermates at E18.5.
10.1186/s13578-016-0108-4 *Otg1* mutation does not affect hepatocyte size. **(A)** H&E staining of liver sections from P11 *Otg1* ^*PB/PB*^ and Otg1^*PB/*+^ littermates. **(B)** Relative hepatocytes size of P11 *Otg1* ^*PB/PB*^ (n = 3) and *Otg1* ^*PB/*+^ (n = 4) littermates.
10.1186/s13578-016-0108-4 GH3 cells co-transfected with VSVG-mEmerald and scramble shRNA.
10.1186/s13578-016-0108-4 GH3 cells co-transfected with VSVG-mEmerald and *Otg1* shRNA-1.
10.1186/s13578-016-0108-4 *Otg1* knockdown blocks vesicle transportation in GH3 cells. **(A)** Unaltered directionality between *Otg1* knockdown (red x) and scramble shRNA treated (black crosses) GH3 cells shown in Fig. 5b. **(B)** Decreased standard deviation of transport speed of cells shown in Fig. 5b. ***p < 0.005.

## Abbreviations

CTCL: cutaneous T-cell lymphoma
GH: growth hormone
GHR: growth hormone receptor
GSIS: islet culture and glucose stimulated insulin secretion
GTT: glucose tolerant test
IDM: The Institute of Developmental Biology and Molecular Medicine
IPGTT: intraperitoneal glucose tolerance test
ITT: insulin tolerance test
MEF: embryonic fibroblast
NICTH: non-islet cell tumor-induced hypoglycemia
*Otg1*: oocyte testis gene 1
*PB*: *piggyBac*
shRNA: small hairpin RNA
TGN: trans-Golgi network
