# Supplementary figures and images for: Disruption of the Golgi protein Otg1 gene causes defective hormone secretion and aberrant glucose homeostasis in mice
Source: Cell Biosci. 2016 Jun 10;6:41. doi: 10.1186/s13578-016-0108-4 (PMC4902905; doi:10.1186/s13578-016-0108-4)

Supplemental Figure 1

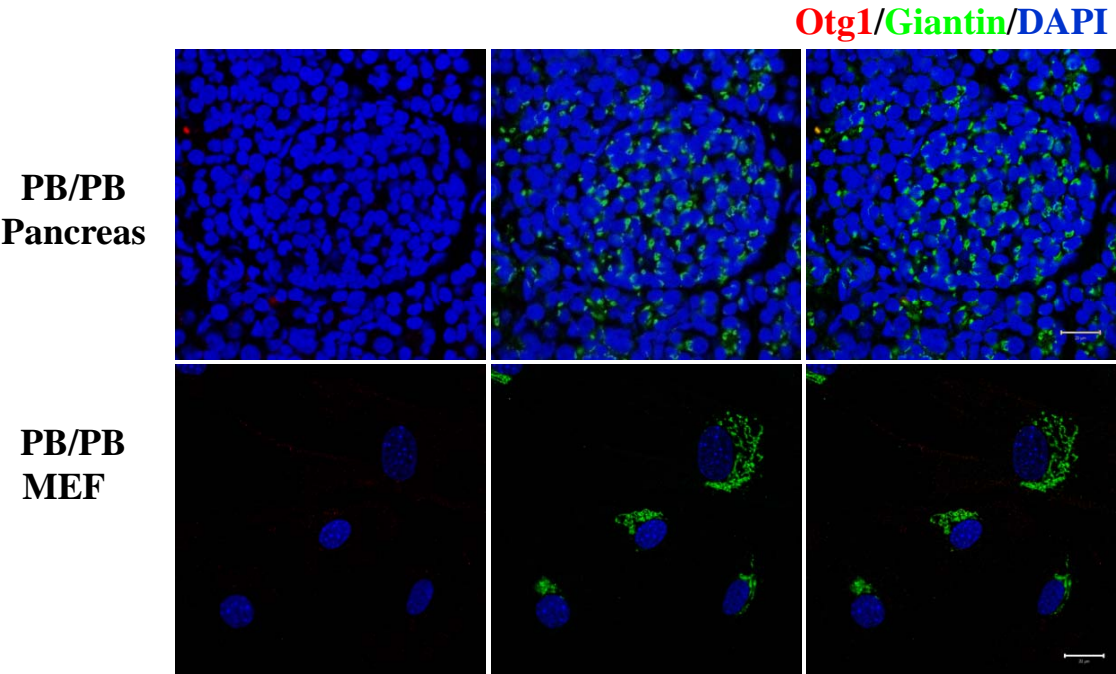

Supplement: Supplementary file 1 — 10.1186/s13578-016-0108-4 Otg1 expression is disrupted by the PB insertion. In contrast to Fig. 1c, immunofluorescence staining showed Giantin (green) but not Otg1 (red) signals in pancreatic tissues and MEFs from Otg1 PB/PB mice. [file 13578_2016_108_MOESM1_ESM.pdf]

Supplemental Figure 2

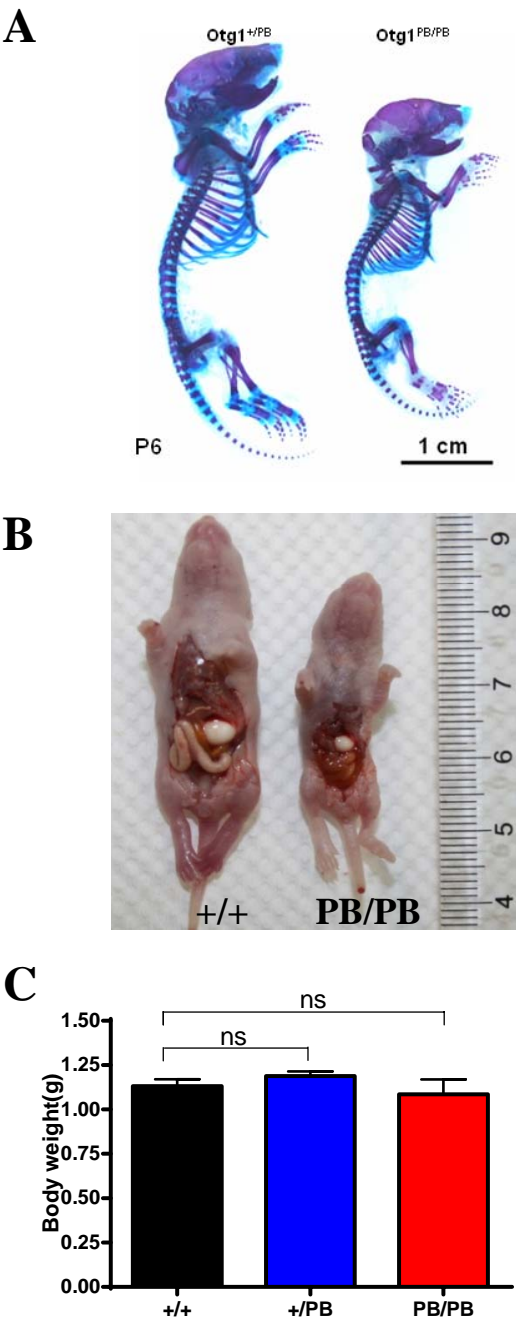

Supplement: Supplementary file 3 — 10.1186/s13578-016-0108-4 Otg1 mutant mice have no severe developmental defects. (A) Alcian blue-alizarin red staining of P6 Otg1 +/PB and Otg1 PB/PB mice. (B) Representative image showing P6 wild-type and Otg1 PB/PB littermates, with sucked milk in the stomach. (C) Average body weight of Otg1 PB/PB (n = 6), Otg1 PB/+ (n = 15) and wild-type (n = 15) littermates at E18.5. [file 13578_2016_108_MOESM3_ESM.pdf]

Supplemental Figure 3

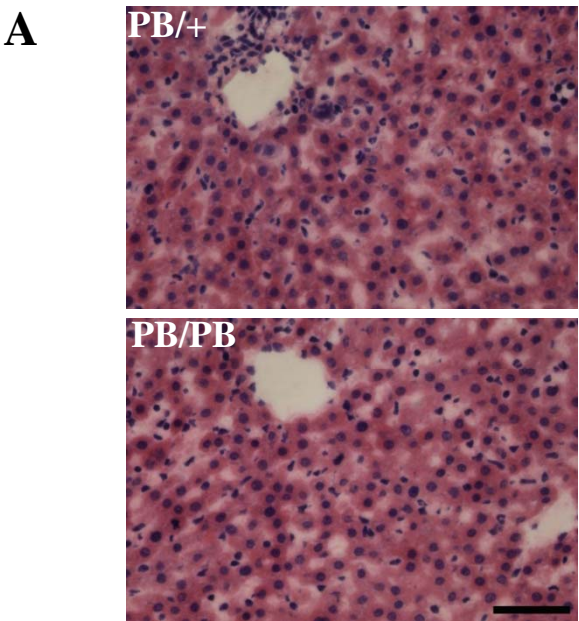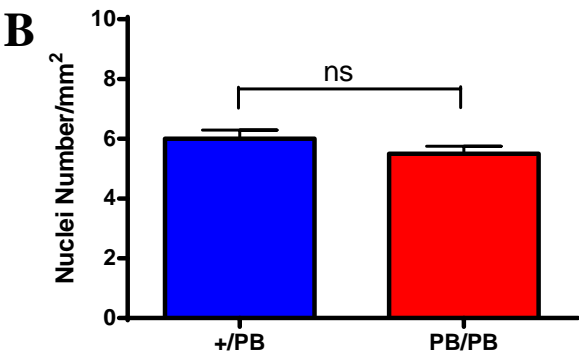

Supplement: Supplementary file 4 — 10.1186/s13578-016-0108-4 Otg1 mutation does not affect hepatocyte size. (A) H&E staining of liver sections from P11 Otg1 PB/PB and Otg1PB/+ littermates. (B) Relative hepatocytes size of P11 Otg1 PB/PB (n = 3) and Otg1 PB/+ (n = 4) littermates. [file 13578_2016_108_MOESM4_ESM.pdf]

Supplemental Figure 4

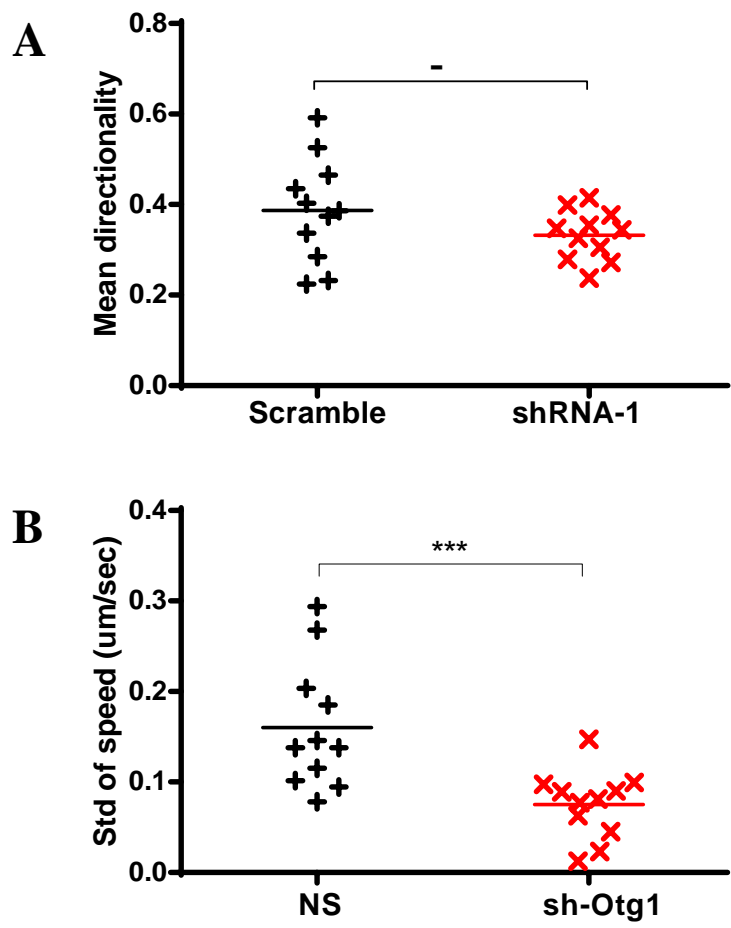

Supplement: Supplementary file 7 — 10.1186/s13578-016-0108-4 Otg1 knockdown blocks vesicle transportation in GH3 cells. (A) Unaltered directionality between Otg1 knockdown (red x) and scramble shRNA treated (black crosses) GH3 cells shown in Fig. 5b. (B) Decreased standard deviation of transport speed of cells shown in Fig. 5b. ***p < 0.005. [file 13578_2016_108_MOESM7_ESM.pdf]
